# Association of Interleukin-10 Methylation Levels With Gestational Diabetes in a Taiwanese Population

**DOI:** 10.3389/fgene.2018.00222

**Published:** 2018-06-22

**Authors:** Jessica Kang, Chien-Nan Lee, Hung-Yuan Li, Kai-Han Hsu, Shu-Huei Wang, Shin-Yu Lin

**Affiliations:** ^1^Department of Obstetrics and Gynecology, National Taiwan University Hospital, Taipei, Taiwan; ^2^Department of Internal Medicine, National Taiwan University Hospital, Taipei, Taiwan; ^3^Institute of Molecular Medicine, College of Medicine, National Taiwan University, Taipei, Taiwan; ^4^Department of Anatomy and Cell Biology, College of Medicine, National Taiwan University, Taipei, Taiwan

**Keywords:** gestational diabetes, interleukin-10, DNA methylation, diabetes mellitus, epigenetics

## Abstract

**Objective:** Gestational diabetes mellitus (GDM) is defined as glucose intolerance with onset during pregnancy, which is also associated with future metabolic diseases in both patients and their offspring. The mechanisms underlying this condition remain largely unknown and may be partly related to epigenetics. The aim of this study was to compare the methylation levels of the cytokine interleukin-10 (IL-10) in pregnant women and their fetuses under both hyperglycemic and euglycemic environments, as those levels may be a clue to the epigenetic mechanisms underlying pathogenesis of GDM.

**Methods:** We analyzed the methylation levels of the IL-10 gene in maternal blood, cord blood, and placental tissue in both a GDM group (*n* = 8) and a control group (*n* = 24) using a LightCycler LC480 (Roche, Rotkreuz, Switzerland). IL-10 concentrations in maternal blood and THP-1 cells were measured by enzyme-linked immunosorbent assay (ELISA) using BD OptEIA Human IL-10 ELISA kits (BD Biosciences Pharmingen, San Diego, CA, United States).

**Results:** The maternal blood IL-10 methylation levels in the GDM group and the control group were 0.23 ± 0.04 and 0.26 ± 0.04, respectively (*p* = 0.03), but there were no significant differences between the levels of the two groups in the cord blood or placental tissue. Increased IL-10 plasma concentrations were discovered under hyperglycemic environments and were confirmed via the THP-1 cell line.

**Conclusion:** Hypomethylation of maternal blood and increased plasma IL-10 concentrations before birth were found in the GDM group.

## Introduction

Gestational diabetes mellitus (GDM) is a metabolic disorder in which insulin resistance develops during pregnancy (American Diabetes Association, 2018), and the population of patients with GDM is rising due to the high prevalence of obesity. An increasing incidence of adverse pregnancy outcomes, such as macrosomia, shoulder dystocia, preeclampsia, and cesarean delivery, have been reported in GDM patients (CDC, 2011; Wendland et al., 2012). In addition, the intrauterine hyperglycemic environment may have a metabolic imprint on the next generation (Aerts and Van Assche, 2006), with the prevalence rates of obesity, metabolic syndrome, impaired glucose tolerance, and future diabetes having been found to be higher in the offspring of GDM mothers (Dabelea et al., 2000; Clausen et al., 2009; Malcolm, 2012). It has also been reported that women with GDM have an increased risk of developing type 2 DM compared with those who have had a normoglycemic pregnancy (Bellamy et al., 2009).

DNA methylation is the most stable epigenetic system and is involved in fetal metabolic and developmental programming (Bird, 2002). Though both animal and human studies have demonstrated an association between GDM and the risk of obesity and future metabolic diseases, the mechanisms underlying this association remain unclear. Epigenetic modifications might be among the mechanisms underlying the association (Vrachnis et al., 2012). More specifically, intrauterine hyperinsulinemia and hyperglycemia may epigenetically alter developmentally important genes to influence metabolic functions and cause increased risks of obesity and type 2 DM in the offspring. Several studies have investigated different genetic methylation models that were previously used for DM in GDM populations (Kirchner et al., 2013; Haertle et al., 2017). Hypomethylation of the adiponectin gene (ADIPOQ) in placental tissue with increased adiponectin levels was noted in a population with high glucose levels and high insulin resistance indexes (Bouchard et al., 2012). Another study reported hypomethylation of the mesoderm specific transcript (MEST) gene in both placenta and cord blood samples in a GDM population (El Hajj et al., 2012). A more recent study investigated early pregnancy maternal blood DNA methylation status in cases of repeated pregnancies, and the authors found different methylation statuses in separate instances of GDM and discovered several novel genes (Enquobahrie et al., 2015). Thus, fetal epigenome modulation by maternal diabetes provides the most reasonable mechanism for the non-genetic intergenerational transmission of the phenotype (Fernández-Morera et al., 2010; El Hajj et al., 2012; Lehnen et al., 2013).

Inflammation is considered to be involved in the pathogenesis of type 2 DM (Pickup, 2004; Al-Shukaili et al., 2013). Many pro-inflammatory cytokines, such as C-reactive protein (CRP) (Hu et al., 2004), interleukin-10 (IL-10) (Brauner et al., 2014), and tumor necrosis factor- α (TNF-α) (Al-Shukaili et al., 2013), play an important role in regulating the innate immune system and have been found to be related to increased risk of type 2 DM in recent studies. GDM shares the common etiologies and pathways of type 2 DM, but few studies regarding inflammatory markers have been published (Retnakaran et al., 2003; Wolf et al., 2003).

IL-10 is a multifunctional anti-inflammatory cytokine produced by T cells, B cells, and macrophages. It also plays an important role in both stimulating and suppressing immune responses (Chagas et al., 2013; Jin et al., 2013). The association of IL-10 with autoimmune disease is well established (Llorente et al., 1994), while its relationship with type 1 DM (Kikodze et al., 2013) and type 2 DM (Thorand et al., 2005; Brauner et al., 2014) has only been preliminarily defined, and its exact role in GDM has not yet been well established. A recent whole-genome study discovered significant methylation differences of the IL-10 gene between maternal blood and cord blood in a GDM population (Kang et al., 2017), while other studies have reported both decreased and increased IL-10 serum levels in hyperglycemic environments (van Exel et al., 2002; Atègbo et al., 2006; Al-Shukaili et al., 2013).

Although previous reports have suggested a relationship between IL-10 and diabetes (Yaghini et al., 2011; Saxena et al., 2013), as well as its complications such as DM nephropathy (Kung et al., 2010; Mahmoud et al., 2016), only a limited amount of data specifying the association between IL-10 methylation and GDM development has been published. We hypothesized that the IL-10 methylation status might be affected by the hyperglycemic environments, further altering IL-10 production, seen in GDM populations. Thus, the current study investigated the IL-10 gene methylation patterns in different specimens from both fetuses and pregnant women, as well as the relationship between IL-10 concentrations and methylation status.

## Materials and Methods

### Patients

This study included a subgroup of 8 GDM cases and 24 cases of healthy pregnancy for methylation analysis, and a total group of 17 women with GDM and 39 healthy pregnant women who served as normal controls for further IL-10 plasma measurements. We collected 10 ml of maternal fasting blood samples during the first (10 to 13 weeks of gestation) and second trimester (24 to 28 weeks of gestation), which provided detailed lab data for fasting glucose levels, HbA1c levels, and lipid profiles. Three milliliters of maternal peripheral blood was collected upon admission before delivery for methylation and ELISA analysis. Another 5 ml of cord blood was collected within 5 min after delivery of the fetus, and random biopsies of 1 cm^3^ of tissue over four quadrants of placenta from the maternal site were performed within 5 min after placenta delivery. All of the above samples were sent for MethyLight analysis.

For all of the above patients, a 75 g oral glucose tolerance test (OGTT) was performed at 24 to 28 weeks of gestation, and GDM was diagnosed then according to the 2010 International Association of Diabetes and Pregnancy Study Groups (IADPSG) guidelines (IADPSG, 2010). In all 17 GDM patients, the diagnosis of GDM was made, on average, at 26 weeks of gestation. In the eight GDM women selected for methylation analysis, the average week of diagnosis was 25 weeks of gestation. All of the GDM patients received education from a dietitian and underwent diet control during the period of later gestation. No additional medication was used for blood sugar control. Clinical information regarding the newborns and any birth complications, including birth body weights and head and chest circumferences, were recorded by pediatricians and nurses after birth.

All the patients were followed from the first trimester of pregnancy to 6 weeks postpartum at the Department of Obstetrics and Gynecology of National Taiwan University Hospital. Informed consent was obtained from each study subject after the nature of the study was fully explained, and the study was approved by the Ethics Review Committee of National Taiwan University Hospital, Taipei, Taiwan (201609020RINC).

### Extraction of Genomic DNA

Maternal peripheral blood and umbilical cord blood samples were collected in ethylenediaminetetraacetic acid (EDTA)-treated tubes at delivery and prepared for buffy coat specimens. The placenta tissues were kept in sterile containers at 4°C immediately after sampling and processed within 6 h of collection, and cytotrophoblasts were isolated from the placentas. Genomic DNA was extracted from each subject’s blood and placental specimens using a QIAamp DNA Micro kit (Qiagen Inc.) according to the manufacturer’s protocol.

### MethyLight Assay

Approximately 2 μg of DNA per sample was bisulfite treated using a MethylCode^TM^ Bisulfite Conversion Kit (Invitrogen, Carlsbad, CA, United States), eluted in 20 μL of sample buffer and then used for MethyLight analysis. The accession number of IL-10 gene used in this study is NG_012088 (GenBank). The target sites of the gene was one CpG located 525 bp downstream of the transcriptional start site for exon 1.

After sodium bisulfite conversion, PCR was performed on a Roche LightCycler LC480 (Roche, Rotkreuz, Switzerland) containing 2 μl of bisulfite treated DNA (200 ng), 2.5 μl of each primer (forward 5′-TTTGGAAAGATTTTAGGGATTAAGAA-3′, backward 5′-AAACTAAACCAAATAATACAATAAA-3′), 0.3 μl of each probe (5′ FAM-TGGAAACGTTTTAAGTAGAGG-BBQ 3′, 5′ YAK-TGGAAATGTTTTAAGTAGAGG-BBQ 3′) and 5 μl of Taq polymerase. The cycling conditions were as follows: 1 cycle at 95°C for 10 min; 40 cycles at 95°C for 10 s, 60°C for 60 s, and 40° for 30 s; and another single cycle of 40°C for 30 s. We obtained both methylated and unmethylated intensities and calculated beta-values manually via equation (1) (by default, α = 100) (Du et al., 2010):

(1) $$\begin{array}{c} \mathrm{Beta}\;\mathrm{value}\;=\;\mathrm{methylated}\;\mathrm{intensity}/(\mathrm{methylated}\;\mathrm{intensity}+\mathrm{unmethylated}\;\mathrm{intensity}+\alpha ) \end{array}$$

### ELISA

Whole blood samples were collected in EDTA tubes, centrifuged, aliquoted into small tubes and stored at -80°C. The IL-10 concentrations of these plasma samples were measured by ELISA using BD OptEIA Human IL-10 ELISA Kit II kits (BD Biosciences Pharmingen, San Diego, CA, United States) according to the manufacturer’s instructions. Repeated freeze-thaw cycles were avoided.

### Effects of Hyperglycemia on THP-1 Cells

The genetic expression of IL-10 is located mostly in human monocytes (Williams et al., 2002). Thus, we chose THP-1 cells, which constitute a human monocytic cell line derived from an acute monocytic leukemia patient obtained from the Bioresource Collection and Research Center (BCRC 60430; Food Industry Research and Development Institute, Hsinchu, Taiwan), for validation of the hypothesis that IL-10 concentrations might be affected by hyperglycemia. Cells were maintained in RPMI 1640 medium supplemented with 2 mM L-glutamine and containing 1.5 g/L sodium bicarbonate, 4.5 g/L glucose, 10 mM HEPES, 1 mM sodium pyruvate, and 10% fetal bovine serum in a 5% CO_2_ incubator at 37°C. Overnight serum-starved cells were divided into two groups and kept under euglycemic (5 mM glucose) and hyperglycemic (16.7 mM glucose) environments. Then, IL-10 concentrations at 0.5, 2, 6, and 24 h were obtained via transformed optical density (OD) values.

### Statistical Analysis

The Mann-Whitney *U* test was used for patient characteristics, methylation levels and ELISA results. Non-normally distributed variables were log-transformed to obtain a normal distribution before statistical analyses were conducted to obtain the geometric mean. The significance level was set at *P* < 0.05 (two-sided). Statistical analyses were performed using SPSS V22.0 software.

## Results

### Patient Characteristics

Characteristics of the study participants are presented in **Tables 1, 2**.

**Table 1:** Patient characteristics of the methylation group.

Prenatal IL-10 methylation group	Normal (*n* = 24)	GDM (*n* = 8)	*P*-value
		
	Mean (*SD*)	Mean (*SD*)	
First trimester			
Maternal age (years)	34.8 (3.6)	38.4 (4.1)	0.09
Height (cm)	158.9 (4.1)	158.2 (4.7)	1
Weight (kg)	56.1 (9.0)	57.2 (9.8)	0.47
BMI (kg/m^2^)	21.8 (3.6)	21.8 (4.0)	0.86
Fasting glucose (mg/dL)	81 (4.4)	91 (6.3)	0.04*
HbA1c (%)	5.3 (0.1)	5.8 (0.2)	0.04*
Fasting cholesterol (mg/dL)	190 (10)	225 (2.1)	0.09
Fasting triglyceride (mg/dL)	129 (42)	180 (82)	0.37
Fasting LDL (mg/dL)	106 (23)	137 (22)	0.07
Fasting HDL (mg/dL)	74 (10)	67 (2.1)	0.55
Second trimester			
Fasting glucose (mg/dL)	77.2 (5.4)	84 (7.2)	0.04*
1 h post-OGTT (mg/dL)	136.3 (18)	179.5 (30)	0.002*
2 h post-OGTT (mg/dL)	118 (18)	141 (27)	0.04*
HbA1c (%)	4.86 (0.26)	5.04 (0.36)	0.032*
Fasting cholesterol (mg/dL)	249 (34)	253 (49)	0.65
Fasting triglyceride (mg/dL)	232 (113)	203 (91)	0.58
Fasting LDL (mg/dL)	81.9 (18)	83.7 (29)	0.69
Fasting HDL (mg/dL)	142 (32)	152 (42)	0.97
Newborn			
Gestational age at birth (weeks)	37.75 (1.6)	38.23 (1.2)	0.64
Birth weight (grams)	2841 (595)	2989 (310)	0.6
Body length (cm)	48.47 (2.2)	49.10 (1.0)	0.95
Head circumference (cm)	33.2 (1.5)	33.7 (1.1)	0.74
Chest circumference (cm)	31.2 (2.0)	31.9 (1.3)	0.6
Shoulder dystocia	*N* = 0	*N* = 0	
Clavicle facture	*N* = 0	*N* = 0	
Hypoglycemia	*N* = 0	*N* = 0	

*^∗^*p* < 0.05.*

**Table 2:** Patient characteristics of the ELISA group.

Prenatal IL-10 ELISA group	Normal (*n* = 39)	GDM (*n* = 17)	*P*-value
		
	Mean (*SD*)	Mean (*SD*)	
Maternal			
Maternal age (years)	33.5 (3.6)	37 (3.7)	0.02*
Height (cm)	161 (5.2)	160 (5.2)	0.42
Weight (kg)	56.2 (8.5)	60.4 (12.2)	0.19
BMI (kg/m^2^)	21.5 (2.9)	23.2 (2.7)	0.089
Fasting glucose (mg/dL)	81.0 (3.8)	81.5 (5.0)	0.036*
HbA1c (%)	5.18 (0.22)	5.33 (0.56)	0.046*
Fasting cholesterol (mg/dL)	171.3 (30.7)	183.3 (51.6)	0.482
Fasting triglyceride (mg/dL)	102.5 (44.6)	144.6 (58.7)	0.096
Fasting LDL (mg/dL)	90.0 (23.7)	100.7 (40.9)	0.567
Fasting HDL (mg/dL)	69.2 (10.8)	64.1 (19.1)	0.639
Second trimester			
Fasting glucose (mg/dL)	77.9 (4.4)	85.1 (11.9)	0.048*
1 h post-OGTT (mg/dL)	129 (24)	178 (40)	0.001*
2 h post-OGTT (mg/dL)	110 (20.3)	162 (26.8)	0.001*
HbA1c (%)	4.85 (0.29)	5.07 (0.44)	0.032*
Fasting cholesterol (mg/dL)	236 (41)	246 (57)	0.83
Fasting triglyceride (mg/dL)	175 (45)	219 (80)	0.135
Fasting LDL (mg/dL)	146 (33)	144 (53)	0.99
Fasting HDL (mg/dL)	89 (32)	78 (22)	0.208
Newborn			
Gestational age at birth (weeks)	39.5 (1.0)	38.5 (1.4)	0.008*
Birth weight (grams)	3168 (323)	3071 (544)	0.54
Body height (cm)	49.9 (1.5)	49.6 (2.3)	0.55
Head circumference (cm)	33.9 (1.3)	33.6 (1.2)	0.52
Chest circumference (cm)	32.5 (1.6)	32 (1.7)	0.49
Shoulder dystocia	*N* = 0	*N* = 0	
Clavicle facture	*N* = 0	*N* = 9	
Hypoglycemia	*N* = 0	*N* = 1	

*^∗^*p* < 0.05.*

#### MethyLight Group

The baseline patient characteristics, including age and body mass index (BMI), were similar between the groups. The fasting plasma glucose, HbA1c levels, and 75 g OGTT results were significantly higher in the GDM group, while no differences between the groups were noted in the first and second trimester lipid profiles. There were also no significant differences in newborn characteristics and birth complications between the groups, which may have been due to the small sample size.

#### ELISA Group

The non-GDM group was younger than the GDM group. The first trimester fasting glucose, 75 g OGTT, and HbA1c results were significantly higher for the GDM group, while there was no difference in either the first or second trimester lipid profile between the groups. There was also no significant difference in fetal birth weight or head circumferences. According to the literature, birth complications such as shoulder dystocia, clavicle fracture, and hypoglycemia are more common in GDM patients. As shown in **Table 2**, there was an elevated incidence of clavicle fracture and hypoglycemia in the GDM group, which may have been due to the intrauterine hyperglycemic environment.

### IL-10 Methylation (MethyLight Assay)

**FIGURE 1:**
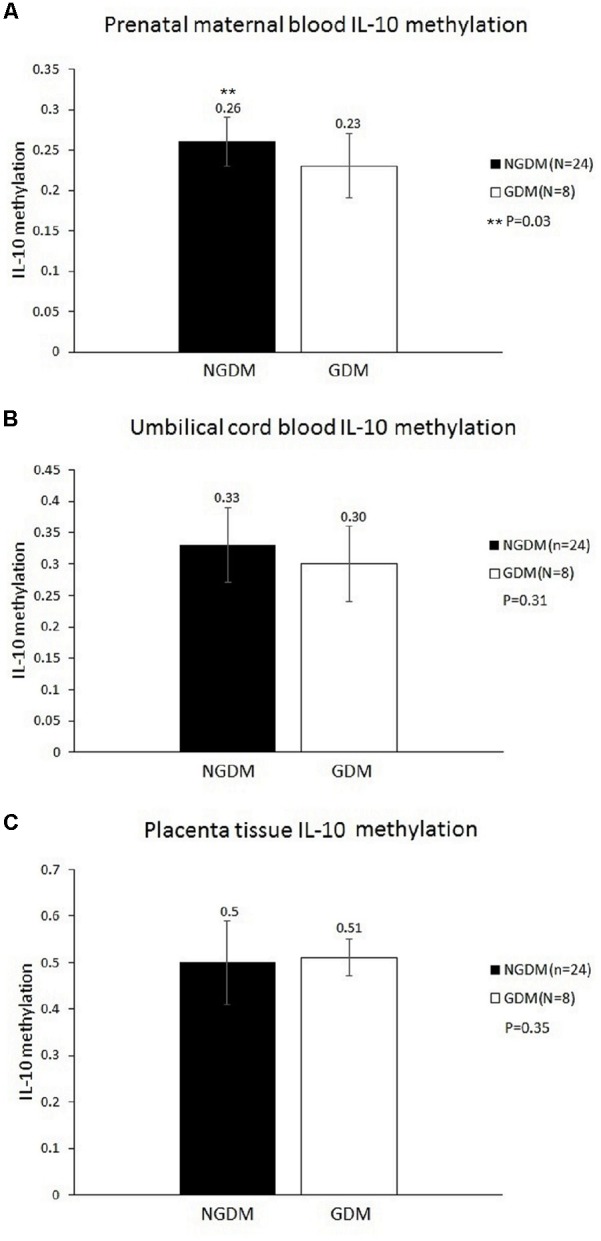
Methylation levels in the prenatal maternal blood, cord blood, and placental tissue. The mean IL-10 methylation levels of maternal blood were 0.23 ± 0.04 and 0.26 ± 0.04 in the GDM patients and the control group, respectively (*p* = 0.03). No significant differences were noted in either cord blood or placental tissue. **(A)** Prenatal maternal blood IL-10 methylation level. **(B)** Cord blood IL-10 methylation level. **(C)** Placenta tissue IL-10 methylation level.

MethyLight was used to determine the methylation and non-methylation intensities. The results showed that the mean IL-10 methylation levels of maternal blood were 0.23 ± 0.04 and 0.26 ± 0.04 in the GDM patients and the control group, respectively, revealing a significantly decreased methylation level in the maternal blood of the GDM group (*p* = 0.03) (**Figure 1**). The mean cord blood IL-10 methylation level in the GDM group was 0.3 ± 0.06, while in the control group it was 0.33 ± 0.07 (*p* = 0.31). The mean methylation levels in placental tissues were 0.51 ± 0.04 and 0.5 ± 0.09, respectively, in the GDM patients and the control group (*p* = 0.35). No significant difference was noted in either cord blood or placental tissue. However, the statistical power values of the cord blood and placenta groups were 0.2 and 0.07, respectively, which might have been the reason for the non-significant results in those two groups. As such, increasing the sample size might provide more satisfactory and reliable results in the future.

### IL-10 Plasma Levels

**FIGURE 2:**
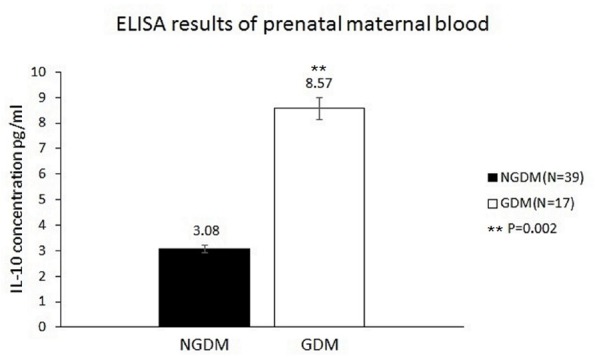
IL-10 concentrations in the prenatal maternal blood. The mean IL-10 concentrations were 3.08 ± 4.68 pg/ml (*n* = 39) and 8.57 ± 7.7 pg/ml (*n* = 17) in the control group and GDM group, respectively. Significantly increased IL-10 levels in prenatal maternal blood were noted in the GDM group (*p* = 0.002).

The IL-10 plasma concentrations were measured by ELISA, and the mean IL-10 concentrations before birth were 8.57 ± 7.7 and 3.08 ± 4.68 pg/ml in the GDM group and the control group, respectively (*p* = 0.002) (**Figure 2**).

### IL-10 Levels in the Hyperglycemic Environment (THP-1 Cells)

**FIGURE 3:**
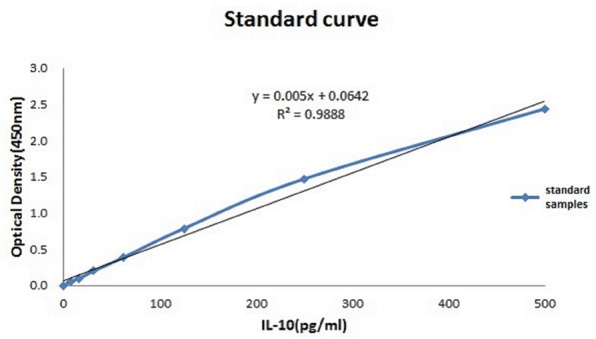
Standardization equation for IL-10 concentrations. The relationship between the absorbance and the IL-10 concentration.

**FIGURE 4:**
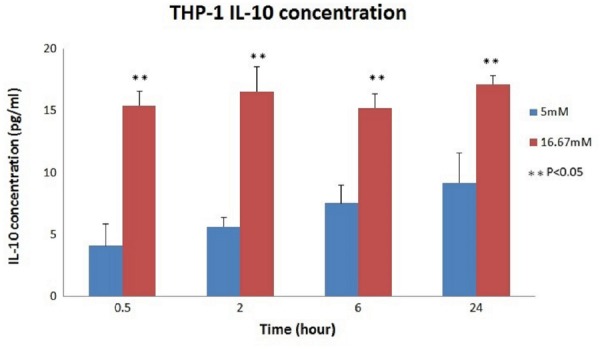
IL-10 concentrations in THP-1 cell. Significantly increased IL-10 concentrations were noted in the hyperglycemic environment in THP-1 cells (0.5 h: *p* < 0.01; 2 h: *p* = 0.02; 6 h: *p* < 0.01; and 24 h: *p* = 0.01).

The relationship between the absorbance and the concentration (pg/ml) of IL-10 was graphed (**Figure 3**); then, the concentration of each group was calculated using the OD values and the equation. The mean IL-10 concentrations at 30 min (15.425 ± 1.172, *p* < 0.01), 2 h (16.558 ± 1.98, *p* = 0.02), 6 h (15.225 ± 1.137, *p* < 0.01), and 24 h (17.158 ± 0.693, *p* = 0.01) in the THP-1 cell line were significantly increased in the hyperglycemic environment (16.7 mM glucose) (**Figure 4**). These results were consistent with the data from our ELISA experiment.

## Discussion

Gestational diabetes mellitus and type 2 DM are both considered to be associated with the inflammatory process. IL-10 is one of the cytokines identified as being related to both type 1 and type 2 DM in previous studies (Thorand et al., 2005; Brauner et al., 2014), and a recent report on a Taiwanese population also documented that IL-10 and IL-4 genotypes are associated with type 2 DM (Shih et al., 2013). To the best of our knowledge, no prior study has investigated IL-10 methylation levels in maternal peripheral blood, cord blood, and placental DNA during pregnancy and how they are affected under hyperglycemic environments. This study investigated this question both epigenetically and molecularly.

We discovered significantly decreased IL-10 methylation levels in maternal blood before birth in the GDM group. The beta-values of the GDM group were all between 0.2 and 0.3, representing average hypomethylation of the IL-10 gene. According to the previous literature, no study has investigated IL-10 methylation levels in a GDM population. The particular timing and mechanism of methylation status remain uncertain and might occur far before pregnancy to determine the fate of a pregnant woman, or they might be induced by pregnancy. Moreover, DNA methylation can silence genes and inhibit their transcription, which may be affected by drug, dietary, and behavioral interventions (Kirchner et al., 2013). All of our GDM patients received diet control only, and none had any need for further medical intervention. That said, methylation status is likely to be altered by slight changes, such as diet control, and the degree of methylation might be more significant when evaluating more severe cases of GDM. A future study could aim to investigate the methylation levels during different trimesters of pregnancy and in cases with differing levels of GDM severity, an approach which might provide a more comprehensive understanding of the actual mechanism underlying IL-10 methylation.

There was no differential methylation in placental tissue noted between the GDM and control groups in our study. Placenta is a hypomethylated human tissue composed of several different cell types that have very different methylation profiles (Grigoriu et al., 2011; Bianco-Miotto et al., 2016). Although some previous investigations discovered differences between healthy controls and women with pregnancy complications in terms of the methylation patterns in their placentas and umbilical cord blood (Ruchat et al., 2013; Finer et al., 2015), another study found no differences in placenta DNA methylation between women with fetal growth restriction and healthy controls, while the same study did find cord blood differences (Hillman et al., 2015). The details of DNA methylation in placental tissues remain largely unknown, and the varying methylation profiles described above might have been the reason that no significant differences were noted in our study.

In our own recent study, whole genome methylation variations in maternal and cord blood between a GDM population and a control group were discovered (Kang et al., 2017). When the analysis is narrowed down to a specific gene such as the IL-10 gene, however, the methylation profile is still uncertain due to a lack of prior studies and inadequate sample size. Limited durations of hyperglycemia before birth might also be one of the reasons that no differences in cord blood methylation status were found.

Fluctuating serum IL-10 concentrations were noted throughout the course of pregnancy in the women in the current study. Increasing IL-10 levels have been noted in early pregnancy, and then the levels decrease at term and increase slightly again after delivery. Increased cortisol levels during pregnancy may be the cause of these fluctuations (Elenkov et al., 2001). When considering the relationship between IL-10 concentrations and GDM, it is important to note that both decreased and increased IL-10 serum levels in GDM populations have previously been reported. One of the studies in question reported relatively low serum IL-10 levels with high HbA1c and high blood glucose levels (van Exel et al., 2002), while other studies showed significantly increased IL-10 serum levels in GDM women and type 2 DM patients (Atègbo et al., 2006; Al-Shukaili et al., 2013). The results of our study were compatible with those of the latter studies that showed increased IL-10 maternal plasma concentrations in GDM patients.

To validate the relationship between plasma IL-10 levels and glucose concentrations, we performed an experiment using THP-1 cells, which constitute a monocytic cell line in which the IL-10 gene can be expressed. The results were compatible with our hypothesis, suggesting that IL-10 levels are strongly related to glucose concentrations in an increased hyperglycemic environment. Our ELISA results for maternal blood before birth also showed significantly increased IL-10 plasma concentrations in the GDM population. The above data support the notion that intrauterine exposure to hyperglycemia has an epigenetic effect on pregnant women. Hypomethylation and increased IL-10 concentrations in the maternal blood before birth were discovered in the GDM group, but no significant differences in the cord blood or fetal placenta were noted.

The limitation of this study was its small sample size, as only 32 cases were included in the methylation study. Upon comparing patient characteristics, no significant difference was identified between the two groups (in terms of lipid profiles, birth body weights, and head circumferences), with the exception of differences in fasting glucose levels, HbA1c levels, and OGTT results. The differences in methylation profiles might be more significant when comparing more severe types of GDM. Though the results revealed decreased methylation levels with increased IL-10 concentrations in maternal blood, we chose to draw the peripheral blood right before birth; this time point represented the methylation status at the end of pregnancy. With regard to preventive medicine, indicators should be noted earlier in the course of pregnancy. Thus, we could change the timing of when the 75 g OGTT experiment is performed to approximately 24 to 28 weeks of gestation or even earlier in the first trimester. Separating the study groups into different trimesters might also provide more thorough evaluations and suggestions in the future.

## Conclusion

IL-10 hypomethylation was discovered in the maternal peripheral blood of GDM patients in comparison to healthy controls, while no significant differences were found in the placenta or cord blood. The IL-10 plasma concentrations were correlated with GDM.

## Author Contributions

JK was contributed to the manuscript drafting and revision. JK, K-HH, and H-YL developed the hypothesis and research question and analyzed and interpreted the patient data. C-NL, S-HW, and S-YL contributed to the data collection and revising the manuscript. All authors read and approved the final manuscript.

## Conflict of Interest Statement

The authors declare that the research was conducted in the absence of any commercial or financial relationships that could be construed as a potential conflict of interest. The reviewer RR and handling Editor declared their shared affiliation.
